# NOVA1 directs PTBP1 to *hTERT* pre-mRNA and promotes telomerase activity in cancer cells

**DOI:** 10.1038/s41388-018-0639-8

**Published:** 2018-12-19

**Authors:** Mohammed E. Sayed, Laura Yuan, Jerome D. Robin, Enzo Tedone, Kimberly Batten, Nicole Dahlson, Woodring E. Wright, Jerry W. Shay, Andrew T. Ludlow

**Affiliations:** 1 School of Kinesiology, University of Michigan, 401 Washtenaw Ave., Ann Arbor Michigan, MI 48109 USA; 2 Department of Cell Biology, UT Southwestern Medical Center, 5323 Harry Hines Boulevard, Dallas, TX 75390 USA; 3 Marseille Medical Genetics (MMG), Aix-Marseille University, UMR125, Marseille, 13385 France

**Keywords:** Chromosomes, Cancer, RNA splicing

## Abstract

Alternative splicing is dysregulated in cancer cells, driving the production of isoforms that allow tumor cells to survive and continuously proliferate. Part of the reactivation of telomerase involves the splicing of *hTERT* transcripts to produce full-length (FL) *TERT*. Very few splicing factors to date have been described to interact with *hTERT* and promote the production of FL *TERT*. We recently described one such splicing factor, NOVA1, that acts as an enhancer of FL *hTERT* splicing, increases telomerase activity, and promotes telomere maintenance in cancer cells. NOVA1 is expressed primarily in neurons and is involved in neurogenesis. In the present studies, we describe that polypyrimidine-tract binding proteins (PTBPs), which are also typically involved in neurogenesis, are also participating in the splicing of *hTERT* to FL in cancer. Knockdown experiments of PTBP1 in cancer cells indicate that PTBP1 reduces *hTERT* FL splicing and telomerase activity. Stable knockdown of PTBP1 results in progressively shortened telomere length in H1299 and H920 lung cancer cells. RNA pulldown experiments reveal that PTBP1 interacts with *hTERT* pre-mRNA in a NOVA1 dependent fashion. Knockdown of PTBP1 increases the expression of PTBP2 which also interacts with NOVA1, potentially preventing the association of NOVA1 with *hTERT* pre-mRNA. These new data highlight that splicing in cancer cells is regulated by competition for splice sites and that combinations of splicing factors interact at *cis* regulatory sites on pre-mRNA transcripts. By employing *hTERT* as a model gene, we show the coordination of the splicing factors NOVA1 and PTBP1 in cancer by regulating telomerase that is expressed in the vast majority of cancer cell types.

## Introduction

Telomerase is a ribonucleoprotein complex that is reactivated in ~90% of cancers, allowing cancer cells to maintain their telomeres and providing them limitless replicative potential [1, 2]. Thus, telomerase reactivation has been characterized as a nearly universal feature of cancer [3, 4]. Telomerase activity is abundant in early fetal/embryonic development but is restricted to low levels in most adult somatic cells [1, 5]. Telomerase consists of two core components, the protein catalytic subunit human telomerase reverse transcriptase (*hTERT*) and the RNA template human telomerase RNA (*hTERC*). The limiting factor for telomerase activity in most human cancers is the expression of full-length *hTERT. hTERT* is a tightly regulated gene with regulatory mechanisms at all levels of gene expression [6–8]. Transcriptional regulation of *hTERT* has been the focus of intense study. More recently there is mounting evidence that transcriptional control is not sufficient to completely repress *hTERT* expression [9, 10]. We have demonstrated that TERT transcriptional regulation is controlled by telomere length through chromatin/epigenetic modifications [10]. Even though transcription of TERT mRNA increased with in vitro aging (i.e., short telomeres), neither telomerase activity or TERT transcripts containing the reverse transcriptase domain were detected, suggesting another level regulation. Post-transcriptional regulation, for example alternative splicing, is also important for regulating telomerase activity [9, 11, 12].

The catalytic subunit of telomerase, *hTERT*, consists of 16 exons and is subjected to alternative splicing resulting in several variants, including the full-length (FL) reverse transcriptase (RT) competent variant. Production of the FL RT competent variant is dependent upon cell context and state. For instance, in transit amplifying stem cells and during early human fetal development, FL *hTERT* and several splice variants are expressed simultaneously. The major isoforms that have been extensively studied in development and in cancer involve the splicing of exons 5–9 (5 exons) which encode for part of the RT domain [8, 9, 11, 13–15]. The FL variant of *hTERT* contains all 5 exons in the exon 5–9 region and is the only isoform with the potential to encode for catalytically active enzymes. The next major isoform + α-β, also known as minus beta, is the result of the skipping of exons 7 and 8 which leads to a frameshift and a premature stop codon in exon 10. Another isoform is −α + β, also known as minus alpha, lacks 36 nucleotides of exon 6 which although in frame, generates a dominant negative RT *hTERT* [11]. The last of the 4 isoforms involving splicing of exons 5 to 9 is −α − β. This variant is a combination of both skipping events of minus beta and minus alpha. While there is a consensus on the importance of the RT domain in telomerase activity, little is currently known about the regulation of alternative splicing that generates RT competent versus RT inactive *hTERT*.

Alternative splicing is regulated by a combination of *cis* and *trans-*acting factors and alternative splicing regulation is known to be aberrant in cancer [16, 17]. In the reverse transcriptase domain of *hTERT* three critical *cis* elements were discovered. Two of the elements are located in intron 6, “Block 6 repeats”, which is a variable nucleotide tandem repeat, and “direct repeat 6” were both found to be critical in the production of the minus beta (−β) containing isoforms. This study also identified a region in intron 8 that was critical in the production of FL *hTERT* [18]. This region called ‘direct repeat 8’ (DR8) is conserved across old-world primates but not among other species such as mice and rats [18]. This is important because smaller, shorter lived mammals regulate telomerase differently from larger, longer lived mammals. Rodents have telomerase expression in somatic tissues while primates, including humans, do not [19, 20]. Thus, we hypothesized that DR8 is likely a region where splicing enhancers and repressors of FL *hTERT* splicing are binding. Since we are interested in factors that promote FL *hTERT* transcripts that can generate telomerase activity, we have focused our efforts to date on elucidating factors that bind DR8 of *hTERT*. We recently described a role for the splicing factor NOVA1 in the promotion of FL *hTERT* splicing, telomerase activity and telomere maintenance by binding to DR8 [21]. During this work, we noticed many polypyrimidine tracts located in and around the region where NOVA1 was binding to *hTERT* pre-mRNA in DR8. This observation led us to hypothesize that the polypyrimidine-tract binding proteins (PTBPs) may also have a role in the regulation of *hTERT* splicing.

In this report, we build upon our model that NOVA1 regulates *hTERT* splicing by acting in conjunction with another splicing factor, PTBP1, in cancer cells to enhance the production of FL *hTERT*. Further, we add the discovery that NOVA1 directs PTBP1 to DR8 and promotes FL splicing to our working model of *hTERT* splicing. These data provide an in-depth mechanistic view of how cancer cells regulate *hTERT* splicing and begin to build a strong foundation for designing a cancer therapeutic approach aimed at repressing telomerase that may contribute to stable cancer remissions.

## Results

### Alternative splicing of *hTERT* as a mechanism of telomerase regulation in larger, long-lived mammals

**Fig. 1:**
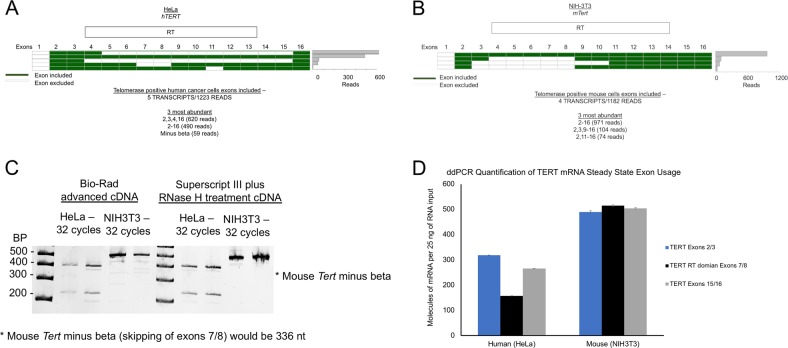
Alternative splicing of hTERT as a mechanism of telomerase regulation in larger, long-lived mammals. Long-read sequencing of *TERT* specific libraries was performed to analyze the splicing isoforms of *TERT* between humans and mice. **a** Human *TERT* libraries were generated with primers in exon 2 and exon 16 and sequenced by long-read sequencing (Pacific Biosciences). A heatmap of included versus excluded exons is shown. A frequency plot displays the number of reads of each isoform that was observed. **b** Mouse *Tert* libraries were generated with primers in exon 2 and exon 16 and sequenced by long-read sequencing (Pacific Biosciences). A heatmap of included versus excluded exons is shown. A frequency plot displays the number of reads of each isoform that was observed. **c** Mouse (NIH3T3) and human (HeLa) cDNAs were amplified with PCR primers spanning TERT exons 5 through 9 of the TERT reverse transcriptase domain (species specific primers were used). *N* = 3 per species, representative image shown. **d** Species specific TERT quantification across the gene body at three different regions of TERT using droplet digital RT-PCR. *N* = at least 6 replicates per primer pair

We have previously identified *cis* elements in human *TERT* (*hTERT)* introns that act as splicing regulatory elements [18]. These *cis* elements, called direct repeat 6 and direct repeat 8 or DR6 and DR8 respectively, were shown to be conserved within old-world primates. Recent work from our lab has built on this regulatory mechanism for *hTERT* alternative splicing by identifying the RNA binding proteins that interact with these regions, in particular DR8 [21]. To determine whether the *cis* acting elements we previously described had a functional role in *TERT* splicing, we compared the splicing of *TERT* transcripts between humans and mice [18]. Humans and other higher order primates have several intronic *cis* elements in the *TERT* gene that seem to regulate alternative splicing, however, rodents and other distant organisms do not have these *cis* elements [18]. First, we evaluated the splicing landscape (i.e., the expressed transcript isoforms from full length cDNAs) of *TERT* RNAs in humans and in mice using targeted long-read length RNA sequencing (long-read sequencing workflow shown in Supplementary Figure 1A). As shown in Fig. 1, human *TERT* is spliced to various transcripts (the 5 most abundant from sequencing reads are shown for the 2–16 libraries from mice and humans). In HeLa cells, full-length *TERT* from exons 2–16 was the second most abundant transcript but only represented 40% of the transcripts in these libraries (Fig. 1a). In contrast, the mouse full-length *Tert* from exons 2–16 comprised 85% of the transcripts sequenced (Fig. 1b, Supplementary Figure 1B and 1D). We prepared human libraries from exons 1–16 (HeLa cells) as well and observed that full-length *TERT* represented about 18% of the total transcripts measured (Supplementary Figure 1C). The difference between the two library preparations among many possibilities could represent either the splicing out of exon 2 (Del2 isoform [22]) as exon 2 was skipped in the 3, 5, and 6 most abundantly detected transcripts, or it could represent the difficulty of the reverse transcriptase and the PCR polymerases to make full-length cDNAs and amplify GC rich regions, respectively. We then looked in depth at the reverse transcriptase domain of human *TERT* and mouse *Tert* (exons 5–9 specifically), since we previously observed *cis* elements in the human intron 8 that appear to be critical for RNA binding proteins (*trans*-factors) that influence splicing of exons 5–9.

Inspection of the mouse *Tert* intron 8 revealed that this species lacks a similar intronic element as compared to human *TERT*. Mouse *Tert* also lacks elements in intron 6 that have been characterized as important for splicing in human *TERT* as well [18]. We used RT-PCR to test for splicing in the region of exons 5–9 and observed that humans generate two prominent splice products (potential full-length *TERT* at 426 bp, Fig. 1c) and minus beta (skipping of exons 7 and 8, 246 bp; Fig. 1c), as expected. When we designed similar primers for mouse *Tert* exons 5–9, we did not observe robust splicing in this region of the reverse transcriptase domain (Fig. 1c). In order to quantify the difference in transcripts containing different combinations of exons across the mouse *Tert* and human *TERT* we designed primers to three different regions. Primers were designed to exons 2 and 3 (5′*TERT*), 7 and 8 (RT domain) and exons 15 and 16 (3′*TERT*) for both mouse and human. Using ddPCR, we observed about a 2-fold difference in total transcripts between human cancer cells (HeLa) and mouse immortal fibroblasts (NIH3T3; Fig. 1d; Average of transcripts measured at all three regions—average ± standard error—human 247.5 ± 47.6 versus mouse 503.2 ± 7.3 molecules of mRNA per 25 ng of RNA input; Student’s *t-*test, *p* < 0.01). These data confirmed that the RT domain of human *TERT* is spliced out of the majority of the transcripts, while in mouse cells the majority of the transcripts contain the RT domain. All together, these data provide strong evidence that the intronic *cis* elements found in humans and other old-world primates act as splicing regulatory regions where *trans*-acting factors likely bind to either promote or repress full-length *TERT* production.

### Basal levels of splicing factors, telomerase activity and *hTERT* splicing in a cell panel

**Fig. 2:**
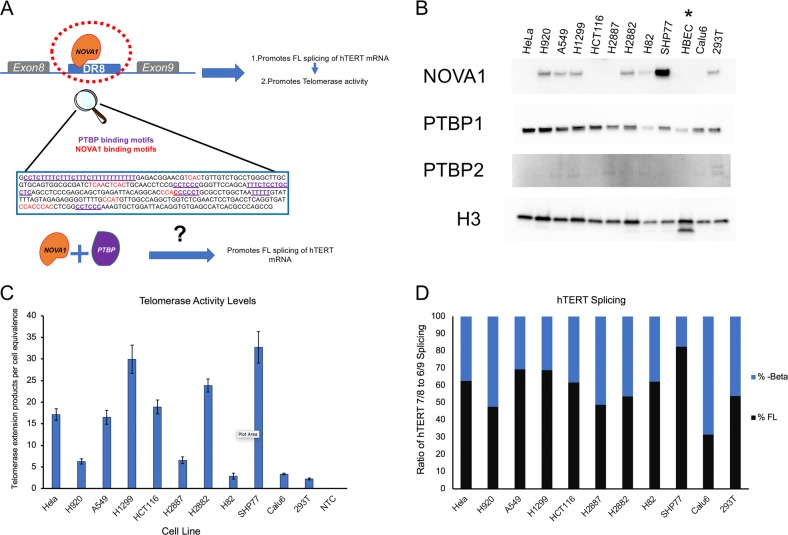
Basal levels of splicing factors, telomerase activity and telomerase splicing in a cell panel. **a** Cartoon model depicting NOVA1 binding to *cis* element DR8 in intron 8. PTBP1 and NOVA1 binding motifs in intron 8 depicted in purple and red font respectively. **b** Western blots for splicing factors NOVA1, PTBP1, and PTBP2 in cell panel. Cell panel consists of normal human bronchial epithelial cells, HBEC, indicated by asterisk as well as a variety of cancer cells. Histone H3 was employed as an internal protein loading control. Repeated three times, representative images shown. **c** Droplet digital TRAP assay detecting telomerase activity using whole cell lysates from the cancer cells in the cell panel (*n* = 3). NTC (no template control) which is used as a negative control. **d** Droplet digital PCR assay quantification of *hTERT* splicing in cancer cells from cell panel. Percentage of FL (exons 7/8) *hTERT* mRNA to −beta (exons 6/9 splicing) *hTERT* mRNA. FL bars are indicated in black and minus beta in blue as indicated in figure legend (*n* = 3). Data are expressed as means and standard error of the mean where applicable

We previously provided evidence that DR8 was a potential binding site for RNA binding proteins [18, 23]. A mini-gene screen targeting RNA binding proteins led to the identification and validation of NOVA1 as a target for regulating *hTERT* FL splicing [21]. NOVA1’s influence on *hTERT* splicing and activity was mediated through its binding of YCAY (Y = C or T) elements within DR8 (Fig. 2a, red text). Upon further examination of DR8’s sequence, we identified an enrichment of polypyrimidine tracts (PPTs, purple text underlined) (Fig. 2a). The PPTs were located in close proximity to NOVA1’s YCAY motifs and even overlapped in one case. Polypyrimidine-tract binding proteins, (PTBs) bind PPTs and regulate mRNA metabolism. PTBs are well known, ubiquitously expressed splicing factors involved in alternative splicing, mRNA stability and translation initiation through its interaction with the ribosome [24]. Humans express 3 homologues (70–80% homology) in a tissue specific manner. The most abundant homologue is PTBP1 which is ubiquitously expressed. The second homologue PTBP2, also known as neuronal PTB (nPTB), is expressed in adult brain, muscle and testis [24]. The third homologue PTBP3, also known as ROD1, is restricted to hematopoietic stem cells [24]. PTBs are known to both activate or repress splicing in a context specific manner [24]. We then tested whether PTBs, in conjunction with NOVA1, bind to DR8 in *hTERT* mRNA and promote the FL splicing of *hTERT* in cancer (Fig. 2a). This model would provide another layer of potential regulation to *hTERT* alternative splicing to further dissect the larger complexity of telomerase regulation.

We first analyzed baseline levels of NOVA1, PTBP1 and PTBP2 in a panel of human tissue culture cell lines consisting of lung epithelial cells (HBEC), non-small cell lung cancer cells (H920, A549, H1299, H2887, H2882, and Calu6), small cell lung cancer (SHP-77 and H82), colon cancer (HCT-116), cervical cancer (HeLa) and transformed embryonic kidney (293T) cells. We observed heterogeneity in the levels of NOVA1 across the cancer lines (7 of 11 tumor cell lines express NOVA1; HBECs did not express NOVA1), with some lines such as Calu6 expressing no NOVA1 and others such as SHP-77 expressing high levels (Fig. 2b). More importantly, no NOVA1 was detected in the human bronchial epithelial cells (HBEC), a model of normal somatic lung cells. This is important because HBECs are non-transformed telomerase negative cells and NOVA1 may be acquired during transformation in certain lung cancers providing them with greater telomere maintenance programs (i.e., more *hTERT* FL). PTBP1 was more readily detected across lines and elevated levels of PTBP1 were observed in the vast majority of cancer lines compared to the normal HBECs. Finally, we observed little to no PTBP2 protein in the panel of cells. The lack of PTBP2 along with elevated levels of PTBP1 in cancer led us to focus our efforts on PTBP1 [25].

We employed droplet digital TRAP and PCR (ddTRAP and ddPCR) to assess levels of telomerase activity and *hTERT* splicing in our cell panel [26]. As expected, we observed heterogeneity in the both telomerase activity and *hTERT* splicing in cancer cells (Fig. 2c, d).

### Stable and transient knockdown of PTBP1 reduces telomerase activity

**Fig. 3:**
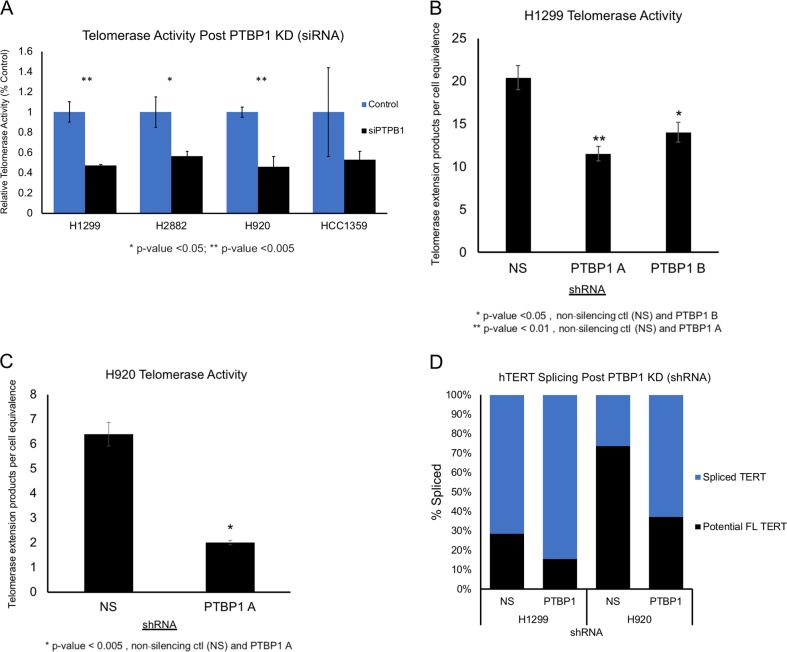
Transient siRNA and stable shRNA knockdown of PTBP1 in non-small cell lung cancer cells, NSCLC. **a** ddTRAP assay detecting telomerase activity post siRNA knockdown of PTBP1 in H1299, H2882, H920, and HCC1359 (50 cell equivalents, *n* = 3, Student’s *t-*test set at *p < 0.05 for significance, **p < 0.005). **b**, **c** shRNA’s targeting PTBP1 were used to generate stable knockdowns in H1299 and H920. Telomerase activity was detected using ddTRAP assay and plotted for H1299 in panel **b** and H920 in **c** (50 cell equivalents, *n* = 3, Student’s *t-*test, *p < 0.05 for significance). **d** Splicing of *hTERT* mRNA was detected and quantified using ddPCR. Spliced TERT, indicated in blue, represents all spliced variants for *hTERT*. Potential FL TERT represents the total steady state *hTERT* mRNAs minus the spliced variants. NS represents non-silencing control shRNAs (*n* = 3). Data are expressed as means and standard error of the mean where applicable. ddTRAP = Droplet digital TRAP

Since PTBP1 is abundant in cancer cells we moved forward with studies focused on PTBP1 over PTBP2. Knockdown (KD) experiments were performed to determine the role of PTBP1 in telomerase regulation and *hTERT* alternative splicing. If PTBP1 was promoting FL splicing of *hTERT*, then knockdown PTBP1 would be expected to result in a decrease in telomerase activity due to the decrease in FL splicing of *hTERT*. We started with transient knockdown of PTBP1 in a panel of non-small cell lung cancer cell lines: H1299, H2882, H920, and HCC1359. We assayed for telomerase activity after cells were incubated with siRNAs targeting PTBP1 [26]. We observed a 50% reduction in telomerase activity in all of the lines (Student’s *t*-test; *p* < 0.05 and *p* < 0.005 as indicated; Fig. 3a). In order to prevent changes in proliferation rates, we used a lower dose of siRNA (10 nM).

Next, we generated stable shRNA mediated knockdown of PTBP1 in both H1299 and H920 cells. We focused on H1299 and H920 lung cancer cells because both lines have robust NOVA1 and PTBP1 expression and showed changes in telomerase activity following transient knockdown of PTBP1 (Fig. 3a). In H1299 cells, we introduced two shRNAs targeting PTBP1 (shRNA A and B). Following PTBP1 knockdown we measured telomerase activity and *hTERT* splicing. Stable KD of PTBP1 significantly reduced telomerase activity in H1299 cells compared to control shRNAs (Fig. 3b), with shRNA A being more potent in reducing telomerase activity. For this reason, we continued our studies focusing only on shRNA A targeting PTBP1. To control for cell line specific phenotypes of PTBP1 targeting shRNAs we introduced shRNA A or control shRNAs into a second NSCLC cell line, H920 cells. Following selection, we observed reduced telomerase activity compared to controls in H920 cells as well (Fig. 3c). Consistent with our telomerase enzyme activity results, upon PTBP1 KD, we found a decrease in the percentage of FL *hTERT* splicing in both H1299 and H920 cells compared to control cells (Fig. 3d; 46% in H1299 cells and 50% in H920 cells).

### Long-term PTBP1 KD leads to an increase in critically short telomeres in lung adenocarcinoma cells

**Fig. 4:**
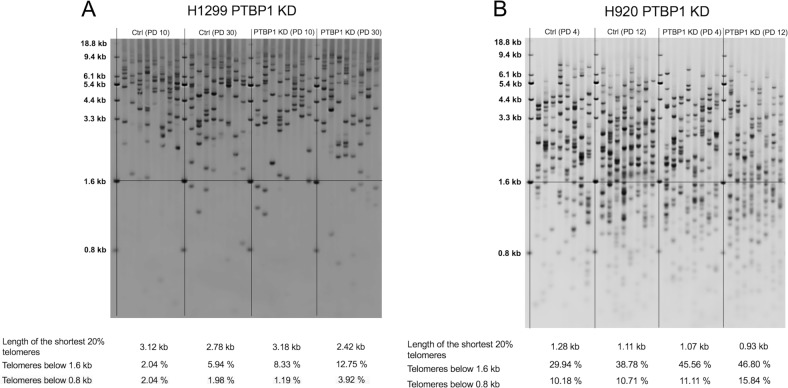
Telomere length analysis in stable knockdown PTBP1 cancer lines. Telomere Shortest Length Assay, TeSLA, was performed to quantitatively assess long-term effects of PTBP1 KD in the cancer lines H1299 and H920. **a**, **b** Stable shRNA H1299 and H920 along with control cells were analyzed with TeSLA. Telomere lengths for both control and PTBP1 KD H1299 cells were measured at population doubling (PD) 10 and 30 (PD10 and PD30). Telomere lengths for both control and PTBP1 KD H920 cells were measured at PD4 and PD12. Data are indicated in the tables below blots. Telomere bands were automatically detected and quantified with accompanying TeSLA software. Each lane represents a separate PCR replicate. In total eight lanes/replicates per sample

In order to determine the impact of long-term knockdown of PTBP1 on telomere length maintenance, we measured telomere length in the stable shRNA PTBP1 KD and control shRNA H1299 and H920 cell lines. **Te**lomere **S**hortest **L**ength **A**ssay, also known as TeSLA, was used to assess the length and percentage of the shortest telomeres [27]. We compared control and PTBP1 KD cells in two different cells lines at similar population doublings post selection. The complete inhibition of telomerase results in the loss of ~50 to 100 nucleotides per cell division. In our experiments we were able to inhibit telomerase by 40–60% in both lines. Given the amount of telomerase inhibition we achieved, we observed the expected progressive telomere shortening overtime in culture. For example, if we assume 100 nucleotide loss per population doubling (PD) in H1299 cells, we would have observed 2000 nucleotides loss of telomere DNA (100 nucleotides × 20 PD). Applying the 40% telomerase activity inhibition due to PTBP1 knockdown (Fig. 3b) to this assumption, we would have expected to observe an 800 nucleotides loss of telomere DNA. We observed a 780 nucleotides loss according to the TeSLA quantification, which is close to the expected amount of telomere shortening. Upon PTBP1 KD, the length of the shortest 20% of telomeres in both H1299 and H920 decreased compared to earlier passaged cells (H1299 PTBP1 KD change of 0.76 kb, Fig. 4a; H920 PTBP1 KD change of 0.14 kb, Fig. 4b). Furthermore, an increase in the percentage of short telomeres, both below 1.6 and 0.8 kb was observed. Although modest, PTBP1 KD in both lung cancer lines increased the critically short telomeres. These data are consistent with the model that PTBP1 KD decreases FL *hTERT* splicing, reduces telomerase activity, and increases the load of short telomeres due to improper telomere maintenance.

### PTBP1 binds *hTERT* pre-mRNA at DR8 via polypyrimidine-tract motifs

**Fig. 5:**
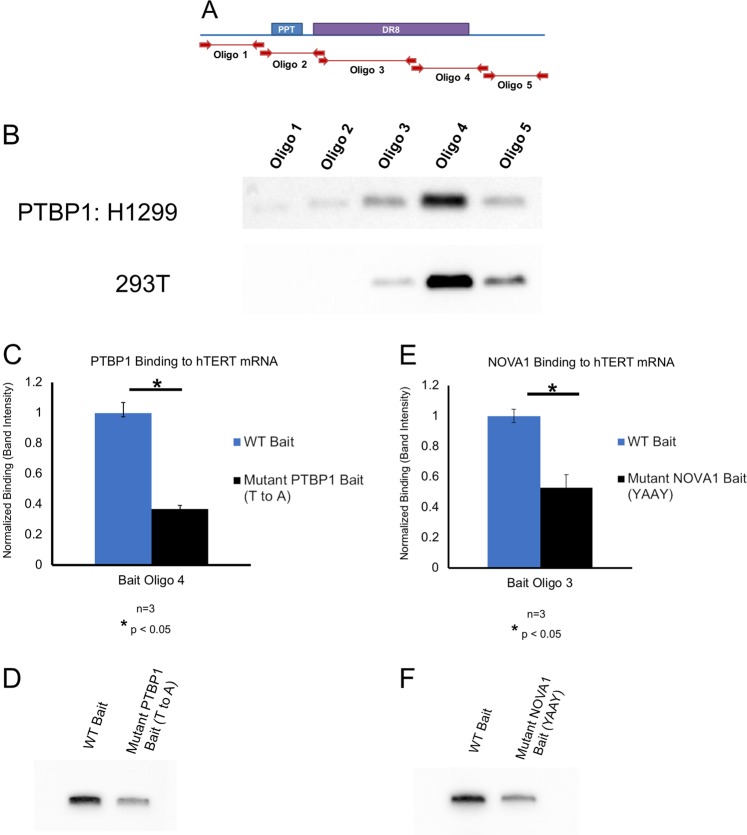
Analysis of PTBP1 and NOVA1 binding to DR8 using RNA baits. **a** Cartoon schematic displaying intron 8 containing *hTERT* pre-mRNA. A large polypyrimidine-tract located upstream of DR8 was included in our analysis. Five oligo baits (oligo 1–5) were designed to encompass 636 nucleotides of *hTERT* intron 8. Baits were then in vitro transcribed into RNA and biotinylated in order to tether on to streptavidin beads. **b** Whole-cell lysates from H1299 and 293T were used to test PTBP1 binding to the five baits. Eluted samples from the RNA-IP were probed via western blot for PTBP1. **c**, **d** Oligo 4 was mutated by altering all T residues to A (U to A in RNA). PTBP1 was then RNA-IP and compared with wild type (WT) bait 4. Western blot probing of PTBP1 showed a reduction in PTBP1 binding to the mutant bait (*n* = 3, Student’s *t-*test set at *p < 0.05 for significance). **e**, **f** Mutant bait was designed for oligo 3 converting all YCAY NOVA1 binding motifs to YAAY. NOVA1 in the elution was probed via western blot and quantified. (*n* = 3, Student’s *t-*test set at *p < 0.05 for significance)

To understand the mechanism by which PTBP1 impacts *hTERT* FL splicing and telomerase activity, we designed a series of RNA baits that mapped in and around DR8 in intron 8 (Fig. 5a). Performing an RNA-IP would allow us to identify the region of intron 8 that PTBP1 binds. Since *hTERT* is a very low abundant pre-mRNA typical cross-linking RNA-IP experiments are difficult to perform compared to analyzing more abundant target pre-mRNAs. To overcome this abundance issue, we turned to a common in vitro RNA-IP pulldown using synthesized RNA baits and cell lysates to probe for proteins that bind to specific RNA sequences. To perform the RNA-IP experiments we transiently overexpressed NOVA1 in 293T cells. NOVA1 binds in oligos 3, 4, and 5, with the strongest binding in oligo 3, as previously shown [21]. Here we show that PTBP1 predominately binds in oligo 4 and to a lesser extent in oligo 3 and oligo 5 (Fig. 5b). Oligo 4 encompasses the last 125 nts of the DR8 region in intron 8. This region did not include the large polypyrimidine-tract that we had expected PTBP1 to have a greater affinity for (the PPT is in oligo 2, Fig. 2a). Interestingly, PTBP1’s binding profile was in close proximity to and to a certain extent overlapped with the binding profile of NOVA1, suggesting the potential for crosstalk or interaction between the two splicing factors.

Next, we performed RNA-IP using mutated baits to further validate the binding of PTBP1 as well as NOVA1 to their respective baits (bait 4 for PTBP1 and bait 3 for NOVA1). For PTBP1 we used the mutant bait 4 in which we replaced all the thymidine bases (T) to adenine (A) disrupting the polypyrimidine tracts (in RNA Uracil (U) to A). As for NOVA1, we mutated the YCAY binding motif to YAAY in bait 3 to generate mutant bait 3, which is known to abolish binding (where Y is C or T/U) [28]. An RNA-IP comparing the sets of control and mutant baits was performed in 293T cells with NOVA1 transiently overexpressed. When we exposed a 293T lysate to a mutant bait 4 (PTBP1 mutant bait 4), we observed a 65% reduction in binding for PTBP1 (Fig. 5c, d). Similarly, when we used a mutant bait 3 (NOVA1 mutant bait 3) we observed a 50% reduction in binding of NOVA1.

### NOVA1 is both necessary and sufficient for PTBP1 binding to DR8 in *hTERT* pre-mRNA

**Fig. 6:**
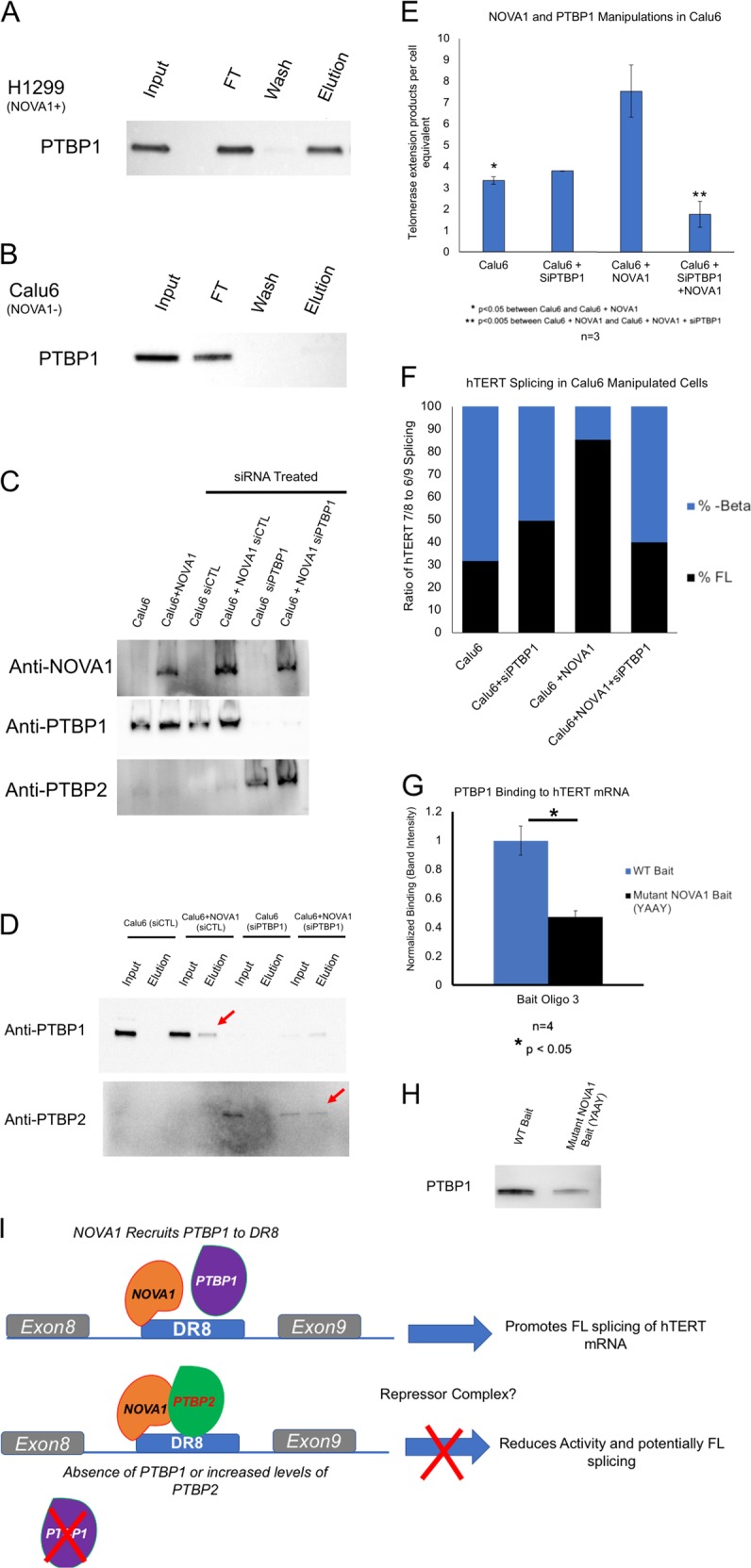
NOVA1 is sufficient and necessary for directing PTBP1 to DR8 in hTERT mRNA. **a** A 1 kb bait of hTERT intron 8 including DR8 was generated to pulldown and detect splicing factors in a NOVA1 expressing cell line (H1299). Elution from RNA-IP was probed for PTBP1 to determine PTBP1 binding to bait. **b** The same 1 kb bait was employed in cell line that does not express NOVA1 (Calu6). PTBP1 was probed for to determine binding. **c** Calu6 lines were manipulated to either overexpress NOVA1, silence PTBP1 (siRNA), or both. NOVA1, PTBP1, and PTBP2 levels were detected via western blot from whole cell lysates. **d** Calu6 (scrambled short interfering RNA control, siCTL), Calu6 + NOVA1, Calu6 + siPTBP1, and Calu6 + NOVA1 + siPTBP1 whole-cell lysates were employed for RNA-IP with RNA bait oligo 4. PTBP1 and PTBP2 levels were probed in input and elution samples for each cell line. Red arrows indicate PTBP1 and PTBP2 binding in elution. **e** Droplet digital TRAP assay was performed on whole cell lysates to determine effects on telomerase activity. (*n* = 3, Student’s *t-*test set at *p < 0.05 for significance, and **p < 0.005). **f** Droplet digital PCR assay to determine FL (exons 7/8) and minus beta (exons 6/9) levels in Calu6 manipulated cells. (*n* = 3) **g**, **h** Quantification and western blot image displaying PTBP1 binding to NOVA1 mutated oligo 3 (YCAY to YAAY) in H1299 whole-cell lysates. Western blot shows elution samples from WT and mutant bait. (*n* = 4, Student’s *t-*test set at *p < 0.05 for significance). **i** Working model cartoon for NOVA1, PTBP1, and PTBP2 action at *hTERT* DR8

Using quantitative assays for telomerase activity and FL *hTERT* splicing pointed out significant heterogeneity in our cancer cell line panel. We leveraged this heterogeneity by comparing cell lines at opposite ends of the spectrum such as Calu6, a low telomerase activity and low *hTERT* FL expressing line, and H1299, a high telomerase activity and high *hTERT* FL expressing line (Fig. 2c, d). Since these two lines display opposite phenotypes with regards to activity and splicing of telomerase, we predicted that they likely have different mechanisms of alternative splicing and potentially employ different sets of *trans-*factors. To test this idea, we designed a 1 kb RNA bait that again encompassed DR8 of *hTERT* pre-mRNA. This bait was then incubated with lysates from Calu6 and H1299 cells (Fig. 6a). We probed for PTBP1, and found PTBP1 was able to bind the *hTERT* pre-mRNA bait in H1299, as seen in the elution, but not in Calu6 (Fig. 6b, c). Since both lines expressed PTBP1 (H1299 slightly more than Calu6) this suggests the presence or absence of a limiting factor in the lysate of Calu6 prevents or allows PTBP1 to bind *hTERT* pre-mRNA. One possibility is that the missing factor may be NOVA1. NOVA1 protein is present in H1299 cells and not in Calu6 cells (Fig. 2b). Secondly, NOVA1’s binding to *hTERT* pre-mRNA was in close proximity and overlapped with PTBP1’s binding (baits 3 and 4). Finally, previous evidence from a yeast two-hybrid screen documented that NOVA1 and PTBP2 proteins interact but it was not known if PTBP1 and NOVA1 interact [29].

Since Calu6 cells normally do not express NOVA1, we expressed NOVA1 in Calu6 cells, Calu6 + NOVA1, to generate a set of cell lines similar besides the expression of NOVA1. To test whether NOVA1 must be present in order for PTBP1 to bind the *hTERT* bait, we transiently knocked down PTBP1 in the Calu6 cells in the presence or absence of NOVA1. We then probed for NOVA1, PTBP1, and PTBP2 levels in the Calu6 cell series (Fig. 6c). NOVA1 was successfully overexpressed and unaffected by siRNA treatments. Furthermore, siRNAs targeting PTBP1 successfully knocked down PTBP1. Previous studies have shown cells upregulate PTBP2 in the absence or depletion of PTBP1 [30]. We observed the same phenomena in the Calu6 lines. PTBP2 protein levels significantly increased upon PTBP1 KD (Fig. 6c).

Calu6 and Calu6 + NOVA1 were treated with control or PTBP1 siRNAs and RNA-IP lysates of each cell line were probed using oligo/bait 4. Oligo 4 was the primary RNA bait bound by PTBP1. As previously shown, PTBP1 from Calu6 siCTL was unable to bind the bait (Fig. 6d). However, after introduction of NOVA1 (Calu6 + NOVA1 siCTL) PTBP1 was now able to bind to *hTERT* pre-mRNA bait (indicated by red arrow, Fig. 6d). This suggests that the presence of NOVA1 is sufficient to direct PTBP1 to *hTERT* pre-mRNA. As expected, the Calu6 siPTBP1 sample displayed little to no binding of the RNA bait (oligo 4). Interestingly, in the Calu6 + NOVA1 siPTBP1 sample, PTBP2 was upregulated and now was able to bind (indicated by red arrow). This was not the case for PTBP2 in the Calu6 siPTBP1 lysate. The presence of NOVA1 was shown to not only direct PTBP1 but also PTBP2 to *hTERT* pre-mRNA bait.

Next, we assessed telomerase activity following transient siRNA mediated knockdown (KD) of PTBP1 (Fig. 6e). NOVA1 was able to increase telomerase activity by increasing the levels of FL *hTERT* mRNA [21]. However, KD of PTBP1 in Calu6 cell line had no impact on telomerase activity compared to siRNA control (Fig. 6e). In the Calu6 + NOVA1 line, PTBP1 KD abolished the increase in telomerase activity compared to Calu6 + NOVA1 siRNA control (Fig. 6e). The decrease in activity can be attributed to an increase in PTBP2 levels. PTBP2 has been previously shown to interact with NOVA1 in mouse neurons [29]. We have also confirmed this interaction via Co-IP in humans (Supplementary Figure 2). Interestingly, we were not able to determine whether or not an interaction between PTBP1 and NOVA1 exists. Taken together, this suggests a potential role for PTBP2 as a repressor complex of FL *hTERT* splicing via direct interaction with NOVA1 at DR8. Percent FL *hTERT* mRNA was reduced by PTBP1 knockdown only in Calu6 cells with NOVA1 expression, similar to the telomerase activity results (Fig. 6f).

The experiments performed above demonstrate that NOVA1 was sufficient to direct PTBP1 to *hTERT* pre-mRNA. In order to determine whether NOVA1 was necessary for PTBP1 binding to *hTERT* pre-mRNA, we employed the mutated oligo 3 in an RNA-IP. Oligo 3 was one of the baits that provided an overlap of binding between the two proteins. By mutating the baits YCAY motifs to YAAY we were able to reduce NOVA1 binding significantly (Fig. 5d, f). If NOVA1 was necessary for PTBP1 binding, then the reduction of NOVA1’s binding to mutant oligo/bait 3 would be predicted to also abolish PTBP1 binding regardless of the polypyrimidine tracts present. RNA-IP using NOVA1 mutant oligo 3 determined that PTBP1 binding was reduced by 50% (Fig. 6g, h). These data provide evidence that NOVA1 is both necessary and sufficient for PTBP1 binding to *hTERT* pre-mRNA at DR8.

## Discussion

Understanding the regulatory mechanisms that govern telomerase activation and activity is critical towards a better understanding of both aging and cancer. Recent evidence has also implicated telomerase in a spectrum of genetic diseases known as “telomeropathies” in which the underlying mechanism is improper maintenance of stem cell telomeres [31], thereby further cementing its importance in biomedical research. In the current study, we focused on the alternative splicing of *hTERT*, a critical yet less understood mechanism of telomerase regulation. We previously reported important *cis* elements, specifically direct repeat 8 in intron 8, that acts as an alternative splicing enhancer of full-length (FL) *hTERT* pre-mRNA splicing [18, 23]. More recently we have identified a role for the splicing factor NOVA1 in binding to DR8 and promoting FL telomerase splicing and activity in non-small cell lung cancer cells [21]. While this has shed new light on telomerase splicing, the process of splicing and the *trans*-acting RNA binding proteins that dictate splicing outcomes is relatively understudied due to the low abundance of *hTERT*. In this report, we build upon the importance of the *cis* element DR8 in intron 8 and the splicing factor, NOVA1 in telomerase regulation with the goal of eventually finding a means to target *hTERT* splicing to promote durable cancer remissions.

The long-read sequencing data of TERT in mouse and human cells reveals a remarkable evolutionary trade-off between telomerase regulation and growth/cancer. The major transcript, approximately 85%, for *Tert* in mice is FL which is capable of producing TERT protein and active telomerase enzyme. In humans however, most *TERT* transcripts are alternatively spliced to inactive variants. Larger, long-lived mammals, such as humans, trade strict regulation on *TERT* through alternative splicing (as well as other mechanisms of regulation) for future reproduction or survival while smaller, short-lived mammals, such as mice, forgo the strict regulations on *TERT* for current reproduction or growth. *TERT* splicing potentially poses as a “current-future” life history trade-off [32]. These types of regulations on *TERT* through alternative splicing also are consistent with Peto’s paradox [33]. The investment into regulatory mechanisms of critical genes, such as TERT, ensures somatic maintenance and allows for larger, long-lived mammals to have lower cancer incidence even though they are made up of more cells that divide over longer periods of time than their smaller, short-lived counterparts [32, 34]. The *cis* element DR8 has a critical role in this regulation. The long-read sequencing data reveals that *mTert* alternative splicing is inherently different than that of human *TERT*. The minus beta isoform in humans, lacking the RT domains exons 7 and 8 necessary for telomerase activity, is not detected in mice. The lack of intronic *cis* elements (both DR6 and DR8) in *mTert* pre-mRNA appears to prevent the splicing of *mTert* in the RT domain. DR8 and other *cis* elements in TERT pre-mRNA are conserved in old-world primates potentially allowing *TERT* to be regulated by alternative splicing via *trans*-acting factors binding to them in those species. The evidence presented provides insights into the importance of DR8 and rationale to further understand the *trans-*factors that bind *hTERT cis* elements in order to determine if manipulation of *hTERT* splicing is a tractable cancer therapeutic approach.

The challenges associated with *TERT* mRNA abundance and the assays used to quantify them have been a significant obstacle. Despite these obstacles, we have reported a novel role for a well characterized and studied neuronal splicing factor, NOVA1 [21]. This led us to believe that there may be other splicing factors regulating hTERT splicing in cancer cells. By identifying additional splicing factors that bind DR8 we can now piece together a more complete picture of *hTERT* splicing. The polypyrimidine tracts in and around DR8 represent an ideal motif for PTBP1 and PTBP2. Using a panel of cells, we confirmed that PTBP2 protein is not present outside of neurons (cancerous and non-cancerous) and thus PTBP1 became the lead candidate gene. Upon transient and stable KD of PTBP1, both telomerase activity and *hTERT* FL splicing were reduced. Furthermore, using the TeSLA assay [27] we were able to report a change in the percentage of short telomeres upon KD of PTBP1 in H1299 and H920 cells, as well as a change in average telomere lengths. We provided evidence that PTBP1 and NOVA1 are indeed binding *hTERT* pre-mRNA sequences. Together, NOVA1 and PTBP1 have a role in driving the FL splicing of *hTERT* in cancer. Interestingly, the data we present here display a master regulator role for NOVA1 as both sufficient and necessary in directing PTBP1 to DR8 in *hTERT* mRNA to promote FL splicing (Fig. 6i). In the absence of PTBP1 (siPTBP1), NOVA1 was able to recruit PTBP2 to *hTERT* mRNA baits. This resulted in a decrease in telomerase activity and FL *hTERT* splicing (Fig. 6f, g). We propose a working model in which the absence of PTBP1 leads NOVA1 to recruit PTBP2 to DR8 and form a repressor complex to prevent FL splicing of *hTERT* (Fig. 6i). This model may have greater implications in cells with elevated levels of PTBP2, such as neurons, where *hTERT* is repressed. Taken together, these data provide significant insights into splicing factor interactions at DR8 in *hTERT* pre-mRNA that may be targeted as a form of therapy in order to disrupt the formation of active telomerase molecules in cancer cells.

## Materials and methods

### Plasmids

GFP pGIPZ shRNA plasmids for control (non-targeting), NOVA1 (Openbiosystems, NOVA1–5′- TTGGACTTAGACAGCTTGA), and PTBP1 (Openbiosystems, PTBP1–5′-TCTGGAAGAACTTGAATCC) were obtained. Lentivirus was made by co-transfecting 5 μg of proviral shRNA plasmids and 2 μg of packaging plasmids pMD2.G and psPAX2 using Polyjet transfection reagent (SignaGen Laboratories) into 293T cells. CCSB-Broad lentiviral human NOVA1 full-length cDNA with a C-terminal V5 tag and blasticidin selection in mammalian cells (accession: BC075038, clone ID: ccsbBroad304_01104) was purchased and sequence verified (GE, Dharmacon). Viral particles were produced as above.

### Cell culture and cell lines

All cancer cell lines (H1299, H920, Calu6, HeLa, A549, HCT-116, H2887, H82, SHP-77, 293T and H2882) were cultured at 37 °C in 5% CO_2_ in 4:1 DMEM:Medium 199 containing 10% calf serum (HyClone, Logan, UT). Briefly, HBECs were maintained in low oxygen conditions in serum free media containing supplements from the Keratinocyte-SFM media (Invitrogen/Gibco catalog # 17005–42) on a collagen/gelatin coated tissue culture dish [35]. All cell lines were obtained from ATCC, or as a kind gift from Drs. John Minna and Adi Gazdar. All cell lines were verified by STR profiling and were tested for mycoplasma contamination.

### Long-read length mRNA/cDNA sequencing

#### Human 2–16 *hTERT* libraries

RNA was extracted from approximately 100 million HeLa cells using the RNeasy plus kit (Qiagen). cDNA was synthesized using two different reverse transcriptases (Superscript III and Bio-Rad Advanced iScript) following manufactures instructions. We use a gene specific priming strategy (5- GTACAGGGCACACCTTTGGT) to generate *hTERT* specific cDNAs with 1000 ng of RNA input. Following cDNA synthesis, fifteen individual cDNAs were pooled and prepared for pulldown using a biotinylated *hTERT* exon 1 oligo (Biotin-5′-AATAATAAT AGCGCTGCGTCCTGCTGCGCACGTGGGAAG). To pulldown *hTERT* cDNAs, the pooled reactions were heated in the presence of 25 μM biotinylated *hTERT* exon 1 oligo to 95 °C for 10 min in a heat block. The heat block was then removed from the heating coil and the block placed on the bench and allowed to cool to room temperature. The cDNAs were then collected with magnetic streptavidin beads (5 μL, Dyna beads). To prepare the beads for binding they were first washed twice in binding buffer (with 0.1% Tween 20 added). Following the beads were blocked in MS2 RNA (0.08 μg/mL MS2 RNA in binding buffer with 0.1% Tween 20) for 10 min at room temperature with rotation. The beads were then collected with the magnet and the blocking solution removed and then added 140 μL of 2×binding buffer. Next, 140 μL of the annealed cDNAs were added to the beads and mixed via a rotator for a minimum of 60 min at room temperature. After the beads were collected by the magnet and washed twice in 1×binding buffer with 0.05% Tween 20. Following the washes captured cDNAs were eluted off the beads by adding 20 μL of 10 mM Tris (pH 8.0) and heated to 85 °C for 5 min and collected immediately on the magnet and the supernatant (cDNAs) collected and placed in a new tube. Following, the exon 1 captured cDNAs were then diluted 1:4 prior to PCR with exon 2 (5′-AAGCATGCCAAGCTCTCG) and exon 16 *hTERT* (5′-AACAATGGCGAATCTGGGGATGGACTATTCCTAT) primers. Following PCR with Emerald AMP GC HS PCR master mix (Takara/Clonetech), 8 reactions were pooled and the DNA purified by phenol:chloroform followed by AMPure beads (Agencourt AMPure XP, Beckman Coulter) to remove primers. An agarose gel was then run and fragments from 3 to 1.5 kb and 1.5 to 0.5 kb were excised from the gel and purified. The samples were then barcoded via PCR to be able to distinguish them following pooled sequencing. Using this strategy, we can assume that all reads we observed contained exon 1. We used this exon 2 strategy because exon 1 of *hTERT* is extremely GC rich and difficult to PCR.

#### Human 1–16 *hTERT* libraries

To generate the human 1–16 *hTERT* libraries the following procedures were performed. We generated gene specific cDNAs as described above with the following modifications. We made 8 cDNAs from HeLa RNA (1000 ng input). The pooled cDNAs were then diluted 1:4 with water. We then performed a first round of PCRs with primers in exon 1 (5′-AGCGCTGCGTCCTGCTGCGCACGTGGGAAG) and exon 16 (5′-AACAATGGCGAATCTGGGGATGGACTATTCCTAT) for 25 cycles using Emerald Amp GC high specificity master mix (8 reactions). The 8 reactions were then pooled and purified with AMPure beads (Agencourt AMPure XP, Beckman Coulter). Following the entire first round of PCR was amplified a second time for 35 cycles with barcoded primers, gel purified into two size bins of 4 to 2.5 kb and 2.5 to 1.5 kb. The size selected barcoded *hTERT* PCR products were then sent to Pacific Biosciences for long-read sequencing on the Isoseq platform.

#### Mouse 2–16 *mTert* libraries

We generated mouse *Tert* exon 2–16 libraries. We observed that exon 1 and exon 2 of mouse *Tert* were similarly expressed and that our focus was on the splicing of the reverse transcriptase domain (exons 4–13) thus we only generated 2–16 libraries for the mouse experiments (Supplementary Figure 1B). To generate the libraries, we isolated RNA from NIH3T3 cells and prepared cDNA using two priming strategies. cDNAs were prepared by inputting 1000 ng of total RNA into the Superscript III (Invitrogen) priming reaction with either a 1:1 ratio of oligo dT:random hexamers (total cDNA) or a gene specific reverse (GSP) primer in exon 16 of mouse *Tert* (5′-TCCGGCACAGCAGTTTTT). Following cDNA synthesis (using manufactures instructions at 55 °C) cDNAs were diluted 1:4 in water. We then prepared 8 PCR reactions with barcoded primers and thermocycled for 40 cycles using Emerald Amp HS PCR mix (Takara). The PCR products were analyzed for size on an agarose gel (5 μL of each PCR). Once PCR size and success was confirmed the remaining PCRs were pooled and cleaned up with AMPure beads ((Agencourt AMPure XP, Beckman Coulter)) to remove excess primers. Samples were then sent for sequencing. Data are deposited and publicly available (accession number-SRP169962).

### Sequence analysis

Following sequence acquisition, data were processed first through quality control and demultiplexing (Pacific Biosciences SMRT analysis software (v1.3.3)). Circular consensus reads (CCS) were counted as reads that had the DNA polymerase pass at least once. SAM files were obtained by aligning FASTQ (of files of the CCS reads) sequences to the genome (either mouse (mm10) or human (hg19)) using GMAP (version 2013-08-19) [36]. Samtools (version 0.1.19) was used to generate BAM and BED files for use in IGV [37]. Data were viewed in IGV and manually counted as primer to primer reads. The plots were generated in R.

### Transient siRNA experiments

For transient knockdown experiments, cells were plated in 6-well plates (150,000 cells per well) and were transfected with non-silencing controls (Santa Cruz Biotechnology, sc-37007) or a pool of three siRNAs targeting PTBP1 (Santa Cruz Biotechnology, sc-38280: sense RNA sequences—(1) 5′-CCAAGAACUUCCAGAACAUtt, (2) 5′-CUUGUGGUAUUACCUUGUAtt, (3) 5′-GCAAUUCCAGGCUCAGUAUtt). Cells were plated 24 h prior to transfections. On the day of transfection, media was switched to 2% serum and transfection complexes were prepared with 50 nM of siRNAs using MEM (Gibco, Invitrogen) and RNAi max (Invitrogen) following manufactures procedures. Following 72 h of exposure to siRNAs, cells were washed, trypsinized, counted and pelleted for downstream assays. For transient transfection assays three biological replicates were completed in technical duplicate resulting in six measures of each condition. These assays were repeated twice in the laboratory.

### Western blot analysis

Total protein lysates were extracted from tissue culture cells using Laemmli buffer, boiled and the protein concentration determined (BCA protein assay, Pierce). Thirty micrograms of protein was resolved on SDS-PAGE gels, transferred to PVDF membranes and detected with a rabbit monoclonal antibody for NOVA1 (Abcam, EPR13847, ab183024, 1:1000 dilution in 5% NFDM), PTBP1 (Abcam, EPR9048B, ab133734, 1:10,000 dilution in 5% NFDM), or PTBP2 (Abcam, EPR9891, ab154853, 1:1000 dilution in 5% NFDM). Protein loading was determined with antibodies against with histone H3 (Anti-Histone H3 antibody produced in rabbit, H0164; Sigma). Blots were imaged with Bio-Rad Chemidoc XRS + Molecular Imager and quantified with Bio-Rad Image Lab software. Analysis shown in Fig. 2b was one cell lysate with the blot repeated twice. Blots shown in Fig. 5b were repeated twice from two separate pulldowns. Figure 5d–f were performed twice in the laboratory with three biological replicates. Figure 6a and b blots were from 1 biological replicated repeated twice in the laboratory. Figure 6c–h are from biological triplicates repeated twice in the laboratory.

### Reverse transcriptase-droplet digital PCR

All cDNAs were diluted 1:4 before use and stored at −80 °C. For *hTERT* splicing analyses we used iScript Advanced (Bio-Rad) to generate cDNAs, diluted 1:4 and used within 48 h of production in ddPCR measures. Primer sequences and methods for calculating percent spliced TERT transcripts for *TERT* are from ref. [21]. Briefly, minus beta, minus alpha, INS3, INS4, and 3′ *hTERT* (exons 15/16) were measured. Total *hTERT* was represented by the summation of INS4, and 3′ *hTERT* (exons 15/16), percentages of the specific splice products calculated, and the remainder assumed to be full length. Individual splice isoform percentages were added together with the percentage of full-length *TERT* and were graphed as percent spliced (Fig. 3d; three biological replicates and two technical replicates). Samples analyzed in Fig. 2 represent three biological replicates of each cell line. Samples analyzed in Fig. 6f are from three biological replicates repeated twice in the laboratory.

### Droplet digital TRAP assay (telomerase activity)

Quantitation of telomerase enzyme activity was performed using a modified telomeric repeat amplification protocol. Briefly, cells were lysed, diluted and added to the telomerase extension reaction for 40 min followed by heat inactivation of telomerase. An aliquot of the extension products was amplified in a droplet digital PCR for 40 cycles and fluorescence measured and droplets read and counted on the droplet reader (QX200, Bio-Rad). Following, data was processed and telomerase extension products per cell equivalents determined. Each droplet digital TRAP assay was repeated in biological triplicate and technical duplicate.

### Shortest telomere length measurement

Genomic DNA was extracted using the Gentra Pure DNA extraction kit (Qiagen) according to the manufacturer’s instructions. Each DNA sample was quantified on a Nanodrop (Thermo Scientific) for concentration and purity, and integrity of DNA was determined as previously indicated [27].

The Telomere Shortest Length Analysis (TeSLA) method was performed exactly as described [27]. Briefly, 50 ng of genomic DNA was added to a final volume of 20 μL of ligation buffer containing 1000 units of T4 DNA ligase (New England Biolabs), 1× Cut Smart Buffer (New England Biolabs), 1 mM ATP and 1 nM of TeSLA telorettes (TeSLA Telo 1–6) and incubated at 35 °C for 16 h followed by heat inactivation at 65 °C for 10 min. After ligation, genomic DNA was digested using a set of restriction enzymes (2 U each of CviAII, BfaI, NdeI, and MseI, New England Biolabs) and then treated with 1 U of Shrimp Alkaline Phosphatase (rSAP, New England Biolabs) at 37 °C for 60 min in a final volume of 50 μl. This mixture was subsequently heat inactivated at 80 °C for 20 min and 10 μL of sample were added to 10 μL of adapter ligation mix (1 μM AT adapter, 1 μM TA adapter, 1 mM ATP, 1× Cut Smart Buffer and 2000 units of T4 DNA Ligase) and incubated at 16 °C for 16 h. After adapter ligation, the sample was heat inactivated at 65 °C for 10 min and subsequently diluted to a concentration of 15 pg DNA/μLl (1:25 dilution). For each sample analyzed we performed eight independent PCR reactions (94 °C for 2 min followed by 26 cycles of 94 °C for 15 s, 60 °C for 30 s, and 72 °C for 15 min) using a total of 25 μL mix containing 30 pg DNA, 2.5 U of FailSafe enzyme (Epicenter), 1× FailSafe buffer H (Epicenter) and 250 nM primers (adapter and TeSLA TP). PCR products were run on a 0.85% agarose gel (1.5 V/cm for 19 h) followed by Southern blot analysis to detect amplified telomeres as previously described [27]. Southern blot images were analyzed by using MATLAB-based software to automatically and accurately detect and size annotate the telomere bands including the percentage of shortest telomeres and average telomere lengths [27]. These southern blot gels were repeated twice in the laboratory.

### RNA pulldown with biotinylated RNA baits

A plasmid was generated (TOPO TA) via PCR from a BAC containing *hTERT* (RP11-990A6, CHORI) using primers that contianed a 1 kb fragment of *hTERT* intron eight including DR8. Following integration into the TOPO TA vector, in vitro transcription was performed using the T7 promoter (Ampliscribe T7 kit, Ambion, Life technologies) following the manufacturer instructions including a 45 min DNase step prior to RNA precipitation. RNA was isolated and biotinylated at the 3′ end (Pierce RNA 3′ end biotinylation kit). Biotinylated RNA was purified with streptavidin beads. Cell lysates were prepared following the kit instructions (Peirce Magnetic RNA-protein pulldown kit). Protein-RNA complexes were immunoblotted for either NOVA1, PTBP1 or PTBP2 following pulldown. To produce the smaller RNA baits, T7 promoter sequences were incorporated into the 5′end of the forward primers of each region of interest in and surrounding *hTERT* DR8. The same procedure was followed as previously described for the 1 kb baits to generate the smaller RNA baits. Furthermore, we generated mutant baits with the same procedure. Due to the low abundance of endogenous NOVA1 in most cancer cells, to perform the NOVA1 pulldowns 293T cells were transfected with V5-tagged NOVA1 cDNA construct using lipofectamine 2000. After 48 h, triplicate samples of 10 × 10^6^ cell were washed, trypisinized, counted, pelleted and frozen at −80 °C until analysis. For PTBP1 pulldowns endogenous PTBP1 was able to be probed because it is very abundant. Sample sizes for each pulldown are reported in the western blot section with each figure represented. Sequences for primers used to generate baits (1 kb, wild type and mutant) are as follows:

**Table:** 

	Sequence (5′−3′)
T7 tagged forward primers for oligos 1–5 (PTBP1)	
PT_1 T7 F LY	TAATACGACTCACTATAGGGCGTATCTGCTTGCGTTGAC
PT_2 T7 F LY	TAATACGACTCACTATAGGCACCAGCAAGGAAAGCCTC
PT_3 T7 F LY	TAATACGACTCACTATAGCGATCTCAACTCACTGCAACCTC
PT_4 T7 F LY	TAATACGACTCACTATAGAGGCTGGTCTCGAACTCCTG
PT_5 T7 F LY	TAATACGACTCACTATAGGCAAGCGTCTCTTAGCAACAGG
Reverse primers for oligo 1–5 (PTBP1)	
PT_1 R LY	GCTTTCCTTGCTGGTGCAGA
PT_2 R LY	CGGAGGTTGCAGTGAGTTGAG
PT_3 R LY	TGGGTGGATCACCTGAGGTC
PT_4 R LY	AAGAGACGCTTGCAGCCTAC
PT_5 R LY	GACAAACAGTGAGAGCAGAATAGC
Primers used for mutant construct (PTBP1)	
Forward primers	
T7 Mut PTBP1 Oligo 3 F	TAATACGACTCACTATAGCGATCTCAACTCACTGCAACCTC
T7 Mut PTBP1 Oligo 4 F	TAATACGACTCACTATAGAGGCTGGTCTCGAACTCCT
T7 Mut NOVA1 Oligo 3 F	TAATACGACTCACTATAGCGATCTCAACTaACTGCAACCTC
T7 Mut NOVA1 Oligo 4 F	TAATACGACTCACTATAGAGGCTGGTCTCGAACTCCT
Reverse primers	
Mut PTBP1 Oligo 3 R	TGGGTGGATCACCTGAGGTC
Mut PTBP1 Oligo 4 R	TTGTGTCGCTTGCAGCC
Mut NOVA1 Oligo 3 R	TtGGTtGATCACCTGAGGTC
Mut NOVA1 Oligo 4 R	AAGAGACGCTTGCAGCC

### Statistics

Unless otherwise noted in methods section, figure legend, or in the results section, pairwise Student’s *t*-tests (two-sided) were used to determine statistically significant differences between group means. For detailed information please see the methods subsections above and/or the figure legends for exact sample sizes and replicates of each experiment. Unless otherwise noted the ‘error’ bars represent standard error of the mean (s.e.m.). Significant differences were accepted at a *p* value <0.05.

## Supplementary information

Supplemental Figures 1 and 2

Supplementary Figure Legends

## Data Availability

Data have been deposited in a public repository https://www.ncbi.nlm.nih.gov/sra/PRJNA506254.
