# Supplementary figures and images for: NOVA1 directs PTBP1 to hTERT pre-mRNA and promotes telomerase activity in cancer cells
Source: Oncogene. 2018 Dec 19;38(16):2937–52. doi: 10.1038/s41388-018-0639-8 (PMC6474811; doi:10.1038/s41388-018-0639-8)

Supplementary Figure 1

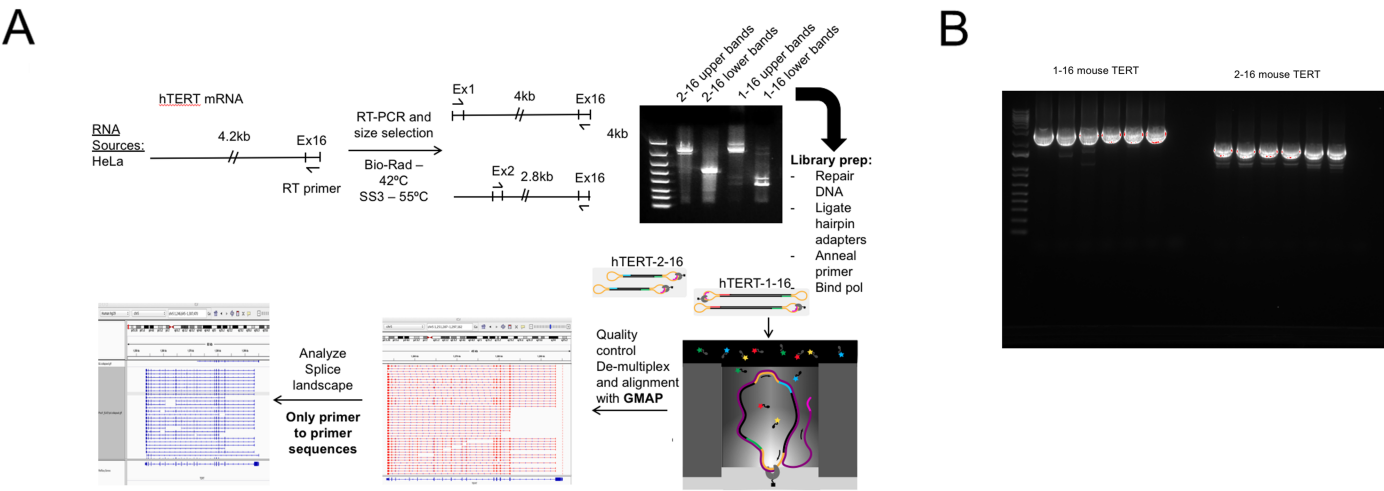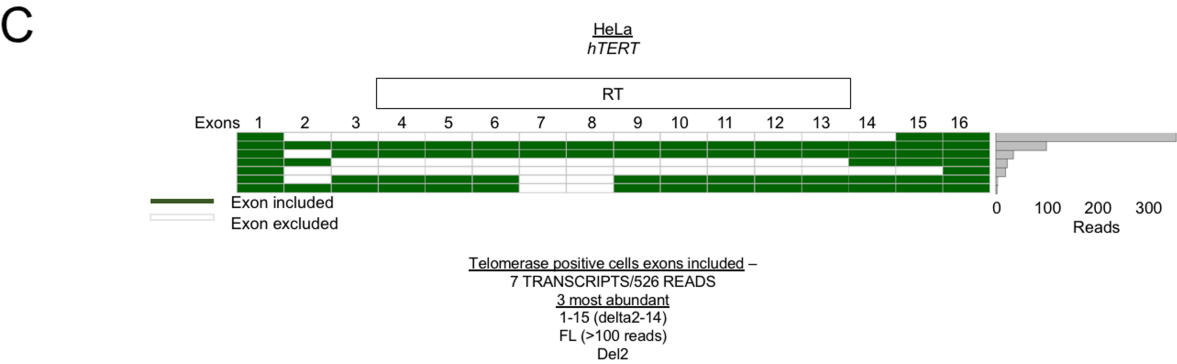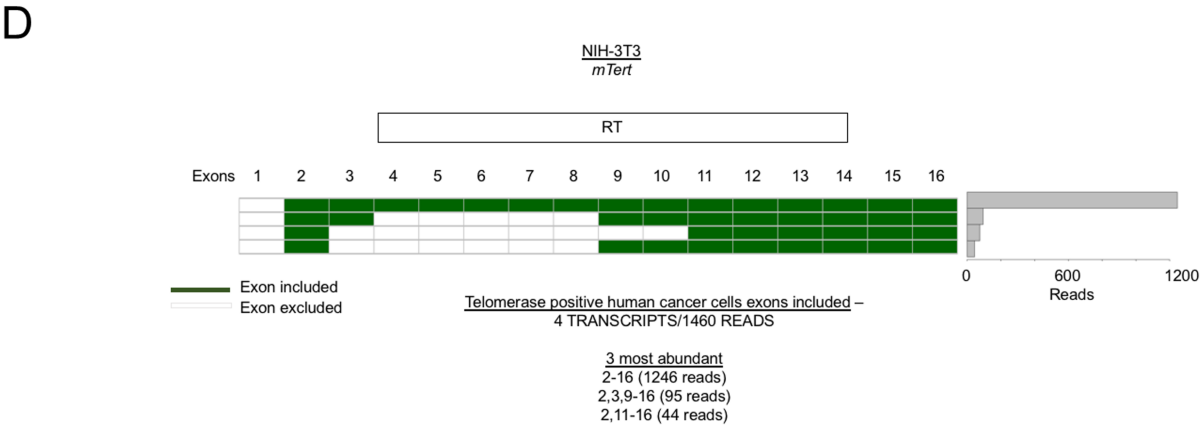

Supplementary Figure 2

293T OE NOVA1 Cross-linked

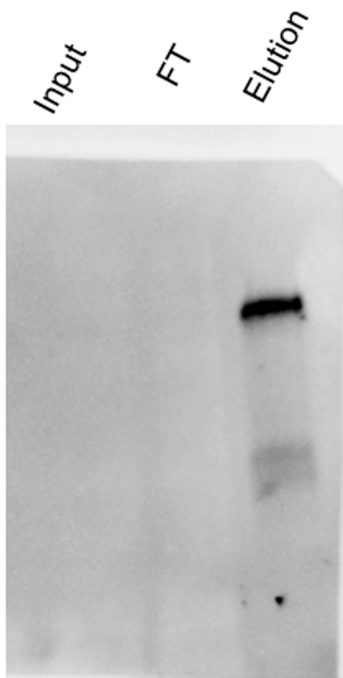

Anti-PTBP2

Supplement: Supplementary file 1 — Supplemental Figures 1 and 2 [file 41388_2018_639_MOESM1_ESM.pdf]
