# Supplementary material for: NOVA1 directs PTBP1 to hTERT pre-mRNA and promotes telomerase activity in cancer cells
Source: Oncogene. 2018 Dec 19;38(16):2937–52. doi: 10.1038/s41388-018-0639-8 (PMC6474811; doi:10.1038/s41388-018-0639-8)
Supplement: Supplementary file 2 — Supplementary Figure Legends [file 41388_2018_639_MOESM2_ESM.docx]

**Supplementary Figure Legends**

**Supplementary Figure 1**: Long-read length sequencing work flow. **(A)** Work flow cartoon showing sources of the human RNA (HeLa cells) and how the gene specific cDNA was made. We used two reverse transcriptases to make cDNA. We then constructed PCR libraries using either an exon 1 or exon 2 primer with an exon 16 primer. Following PCR and barcoding, libraries were size selected into specific pools and sent for sequencing. For sequencing, hairpin adapters were added to PCR products prior to sequencing. Data were then processed, de-multiplexed and aligned to the genomes (human or mouse). We visualized the reads with IGV and counted primer to primer full length reads (circular consensus reads-CCS reads) and recorded the isoforms observed. **(B)** Agarose gel image of mouse TERT amplified with exon 1 and 16 primers and exon 2 and 16 primers. **(C)**. Splicing landscape of *hTERT* following sequencing and data analysis of exon 1 to 16 libraries.

**Supplementary Figure 2:** PTBP2 Co-IP with NOVA1 in 293T + NOVA1 cells. Western blot probing for PTBP2 post crosslinking with NOVA1 in 293T cells that overexpress NOVA1.
